# Pleistocene epilithic foraminifera from the Arctic Ocean

**DOI:** 10.7717/peerj.7207

**Published:** 2019-06-21

**Authors:** Anna Waśkowska, Michael A. Kaminski

**Affiliations:** 1 Faculty of Geology, Geophysics, and Environmental Protection, AGH University of Science and Technology, Kraków, Poland; 2 Geosciences Department, King Fahd University of Petroleum & Minerals, Dhahran, Saudi Arabia

**Keywords:** Biodiversity, Taxonomy, Paleontology, Foraminifera, Pleistocene, Agglutinated Foraminifera, Sclerobionts, Arctic

## Abstract

Attached epilithic foraminifera constitute an important but overlooked component of the benthic foraminiferal assemblage in the Pleistocene sediment of the central Arctic Ocean. We report 12 types of epilithic foraminifera that have colonised lithic and biogenic grains found in glacial sediments, including representatives of the genera *Rhizammina, Hemisphaerammina, Ammopemphix, Diffusilina, Subreophax, Placopsilina, Placopsilinella, Hormosinelloides* and *Tholosina,* accompanied by mat-like and ribbon-like forms of uncertain taxonomic affinity. The attached agglutinated forms appear to be colonisers, adapted to extremely oligotrophic conditions.

## Introduction

A variety of microhabitats and substrate preference is observed among benthic foraminifera. These organisms can settle the sediment surface, live at depth within the sediment, and also attach themselves to elevated microhabitats. Epifaunal and infaunal forms are motile and active, but sessile and attached forms can also be found. These attached forms include those that live attached to pebbles or hard substrate, and in the photic zone some foraminifera attach themselves to plants as well as other hard biogenic objects such as shell fragments, or even other foraminifera. Such forms have been termed sclerobionts by Taylor & Wilson (2002) and Taylor & Wilson (2003). Among the sclerobionts, some genera of foraminifera settle permanently on a hard, presumably stable substrate. Attached agglutinated foraminifera are known from shallow-water to abyssal settings in the modern ocean (e.g., Jones & Parker, 1860; Brady, 1884; Earland, 1934; Altenbach, Unsöld & Walger, 1988; Gooday et al., 1992; Gooday, Goineau & Voltski, 2015; Kuhnt, Collins & Scott, 2000; Waśkowska, 2014; Amao, Kaminski & Frontalini, 2016; Waśkowska & Kaminski, 2018; Förderer & Langer, 2018; and references therein). Such forms usually comprise only a small proportion of the total fauna, but they may become frequent under certain conditions, such as on dropstones or manganese nodules (e.g., Wollenburg, 1992; Resig & Glen, 1997; Gooday, Goineau & Voltski, 2015). The majority of the attached foraminifera in the polar seas and on the abyssal plains belong to organic-walled or agglutinated genera. Many possess fragile tests that are rarely preserved as fossils, consequently their stratigraphic record is poorly known.

In coarse-grained Pleistocene sediments from sediment cores collected on the Lomonosov Ridge during the Expedition 87 of the R/V Polarstern, we observed a variety of sessile agglutinated foraminifera that have attached themselves to larger lithic or biogenic grains. The purpose of the current study is to provide an inventory of the types of epilithic foraminifera observed, and comment on their paleoecology and substrate preferences.

### Study area

**Figure 1:**
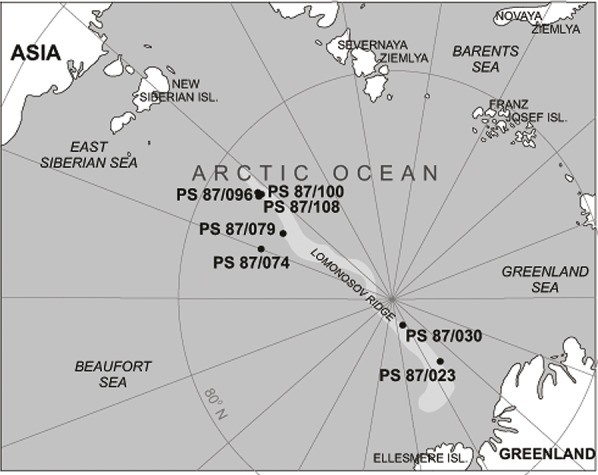
Map of the Arctic Ocean area with the position of studied samples in the area of the Lomonosov Ridge.

Our study area is located in the central part of the Arctic Ocean on the Lomonosov Ridge, a sliver of continental crust that rifted away from Siberia during the early Paleogene (Fig. 1). Sediment cores were collected during Expedition 87 of the R/V Polarstern (the ALEX Expedition) during the summer of 2014 (Stein, 2015). The list of cores from which we observed attached agglutinated foraminifera is given in Table 1. The middle Pleistocene interval, between MIS4 and MIS19 (Stein, 2015) consists of medium brown and dark brown silty clay interbedded with sandy clay. At all sampled sites the Pleistocene sediment is unlithified, and contains ice-rafted material. Calcium carbonate is mainly restricted to the interglacial sediment in the upper intervals of the cores, while sediment deposited during the glacial periods is mostly non-calcareous.

**Table 1:** List of cores collected during the PS87 Expedition in which attached agglutinated foraminifera were found.

Sample	Latitude	Longitude	Water depth
PS87/030-1 kastenlot	88°39.72′N	61°32, 52′W	1,276.8 m
PS 87/074 core catcher	82°43.12′N	158°36.88′E	2,772 m
PS87/023-1 kastenlot	86°38.23′N	44°53.98′W	2,444.8 m
PS87/079-1 kastenlot	83°12.06′N	141°22.77′E	1,360.8 m
PS87/100-1 core catcher	81°21.42′N	142°35.46′E	951 m
87/108–1 core catcher	81°12.79′N	141°10.70′E	1,439.6 m
PS 87/96-1 core catcher	81°12.73′N	141°20.04′E	1,071.4 m

## Methods

Samples were collected at 1-cm intervals from the kastenlot cores, and gently washed through a 63 µm sieve under running water on board ship. Residues were oven-dried at 60 °C, transferred into vials, and foraminifera were picked into cardboard slides under a binocular microscope. Altogether 544 samples from 4 kastenlot cores and 28 core-catcher samples from gravity cores were processed during the PS87 expedition. The attached foraminifera were separated from the picked samples and are the objects of this study. Specimens were photographed in the SEM Laboratory of the Faculty of Geology, Geophysics and Environmental Protection and in the Department of General Geology and Geotourism, AGH University of Science & Technology, Kraków.

## Results

### Attached foraminifera

The mid-Pleistocene sediments on the Lomonosov Ridge contain a benthic foraminiferal assemblage consisting of calcareous and agglutinated foraminifera. The calcareous foraminifera are mostly confined to the most recent interglacial horizons, while the agglutinated foraminifera are found throughout the section in both interglacial and glacial deposits. Among the agglutinated foraminifera we observed a total of 12 types of attached forms that lived on a hard substrate. The occurrences of attached foraminifera in the PS87 samples are given in Table 2. The taxonomy of these forms is still poorly known, and they are described as follows:

**Table 2:** Attached agglutinated foraminifera found in core samples from the PS87 Expedition.

	PS 87/30-01 225–221 cm	PS 87/30-01 234–236 cm	PS 87/30-01 257–259 cm	PS 87/30-01 278–280 cm	PS 87/30-01 295–297 cm	PS 87/30-01 322–324 cm	PS 87/30-01 332–334 cm	PS 87/30-01 354–356 cm	PS 87/30-01 404–406 cm	PS 87/30-01 420–422 cm	PS 87/30-01 437–439 cm	PS 87/30-01 457–459 cm	PS 87/30-01 476–478 cm	PS 87/30-01 499–591 cm	PS 87/30-01 510–512 cm	PS 87/30-01 520–522 cm	PS 87/30-01 536–538 cm	PS 87/30-01 583–585 cm	PS 87/30-01 612–614 cm	PS 87/30-01 zipfelmutze	PS 87/023-1 kastenlot core ccher	PS 87/100-1 0–2 cm	PS 87/079-1 410–412 m	PS 87/079-1 kastenlot core cscher	PS 87/108-1 core cacher	PS 87/096-1 core cacher	PS 87/074-3 core catcher
*Ammopemphix hemisphaericus*		C	C	F	F		C	F	C				C	F	C		F	F			F	C	F		R		
*Diffusilina-* like form																						R					
*Hemisphaerammina* sp*.*			F	R		R		F	R	R	R		C		R	F	R	R	R	F		F	R	R		C	
*Hormosinelloides-* like form																						R					
*Iridia-* like form																						R					
Mat-like form																						R					
*Placopsilina* sp.																						F					
*Placopsilinella auriantiaca*	R	R		F		C			R		R	R					R					R					R
*Rhizammina-* like tube																						R					
Ribbon-like form																						R					
*Subreophax-* like form																						R					
*Tholosina-* like form			R		R												R					F					

**Notes.**

R rare F few C common

**Figure 2:**
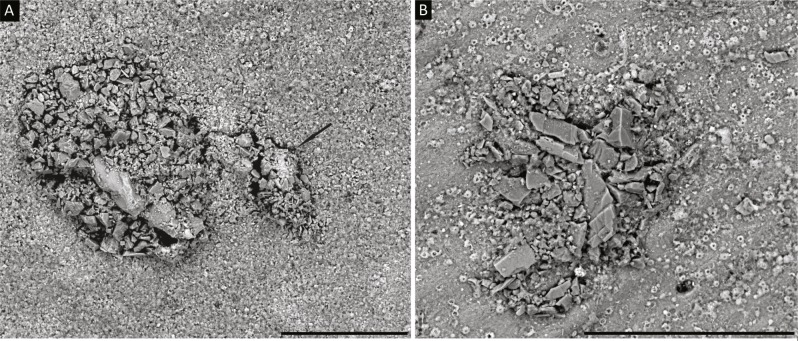
(A, B) *Diffusilina*-like forms attached to the specimen of *C. striolatum*. Scale bars = 100 µm. On A, a small *Tholosina* is attached to the *Diffusilina*-like form, indicated by the arrow.

*1. Diffusilina-* like forms: Test attached, possessing a flat, medium to coarsely agglutinated test wall with occasional larger grains, forming a roof-like structure that covers a depression in the substrate (Figs. 2A and 2B). The structure is even with the surface of the substrate or forms a slight elevation. Specimens vary in dimensions (100–200 µm) and have an irregular outline that conforms to the outline of the depression in which the organism lives. The species selects angular agglutinated grains, and has a rough surface. Aperture not visible. The grains that comprise the test do not form a continuous layer, as between the grains there are pores of various dimensions. The preserved agglutinated layer was probably attached to an inner organic lining. *Diffusilina*-like forms are observed on a specimen of the miliolid foraminifer *Cornuspiroides striolatum* (Brady).

The genus *Diffusilina* Heron-Allen & Earland, 1924 was originally described from Lord Howe Island in the South Pacific. *Diffusilina* was also reported (but not illustrated) from the Weddell Sea by Cornelius & Gooday (2004), and from the northeastern USA by Todd & Low (1981). *Diffusilina humilis* Heron-Allen and Earland, 1924 was reported from the shallow waters of New Caledonia by Debenay (2012). It is a poorly known form, rarely reported in the literature, but it appears to be more widely cited from high-latitudes.

**Figure 3:**
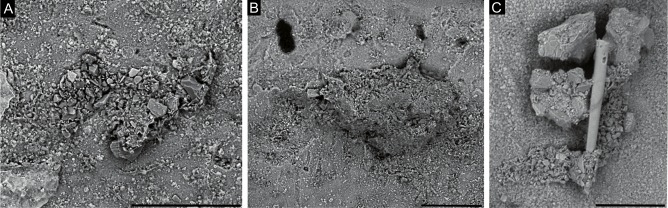
(A) Mat-like form, (B) *Iridia*-like form, (C) ribbon-like form attached to the specimen of *C. striolatum*. Scale bar = 100 µm.

*2. Iridia*-like forms: Test attached, a flat, fine to medium agglutinated. The test is in the form of a 20 µm thick cover. It has an irregular shape, 100–200 µm in diameter, with stolons that radiate out in different directions. In our specimen these are only partially preserved and only the basal portions are present (Fig. 3B). Aperture indistinct. *Iridia*-like form is observed on the specimen of *C. striolatum*.

The genus *Iridia* Heron-Allen & Earland, 1914 was originally described as from the Kerimba Archipelago. Our specimens are most similar to *Iridia diaphana* Heron-Allen & Earland, 1914, but differ in possessing a smaller and more coarse agglutinated grains in the test wall. Only the agglutinated layer of the test has fossilization potential, as the inner organic lining is not present. The genus *Iridia* has been subsequently recorded from the shallow waters of the Mediterranean (Murray, 1991; Meric et al., 2014), the Atlantic Ocean (Cushman, 1918; Debenay, 2000), the Caribbean (Richardson, 2006; Sen Gupta & Smith, 2010), and the Pacific Ocean (Hayward et al., 1999).

*3. Mat*-like forms: Test attached, flat, generally medium-agglutinated, in the form of a mat-like covering of irregular shape on the substrate surface (Fig. 2A). Dimensions 100–150 µm, aperture not visible. The test surface is rough, with occasional larger agglutinated grains. The test is similar to the *Diffusilina*-like forms, but the grains do not form a continuous sheet—they are loose. The agglutinated grains were likely attached to an organic membrane that is no longer preserved. The mat-like forms are observed on the specimen of *C. striolatum*.

Primitive mat-like attached forms of uncertain taxonomic affinity have been noted from deep-water settings. They are often observed attached to manganese nodules in the Pacific Ocean (Mullineaux, 1987; Gooday, Goineau & Voltski, 2015).

*4. Ribbon*-like forms: Test attached, a flat, generally medium- to finely agglutinated, test in the shape of a meandering ribbon, ca. 50 µm wide (Fig. 3C). Only fragments were found, the longest of which is 300 µm. The test surface is rough, comprised of angular quartz grains. The grains are loose—They were likely attached to an organic membrane that is no longer present. The ribbon-like forms are attached directly to the substrate, but may also cover other structures that are attached to the substrate. The ribbon-like forms are observed on the specimen of *C. striolatum*.

**Figure 4:**
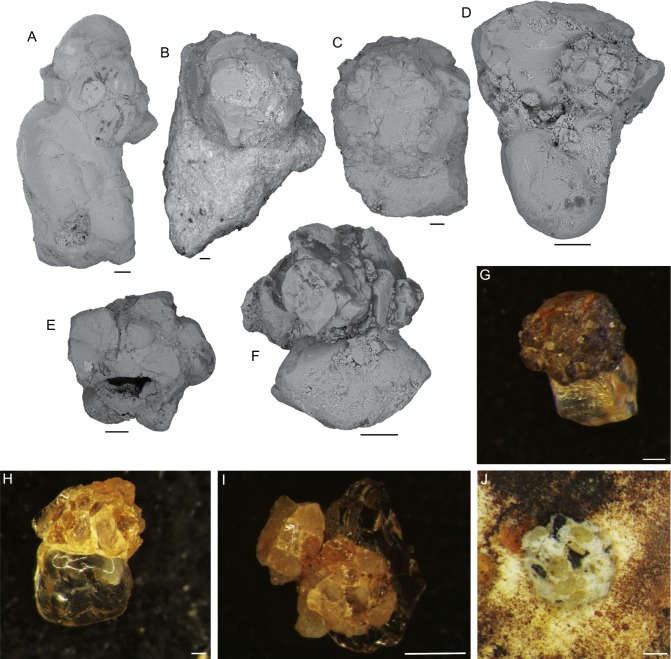
*Hemisphaerammina.* sp. (A–D), (F–I) Attached to the attached to a clastic grain (A—on the lower part of the clast there is a small pseudocolony of *Ammopemphix hemisphaericus* Waśkowska and Kaminski), E—separated test, J—attached to the specimen of *C. striolatum*. Scale bar = 100 µm.

*5. Hemisphaerammina* Loeblich and Tappan, 1957: Test attached, a single large spherical or subspherical to slightly oval chamber attached mostly to large quartz grains (Fig. 4). The wall is coarsely agglutinated, with larger grains surrounded by a matrix of smaller grains. The finely agglutinated matrix acts as the attachment to the larger grain. The species does not possess an agglutinated floor. Loeblich & Tappan (1957) defined the genus as follows “Test attached, a single hemispherical chamber that may have a bordering flange; wall agglutinated, with considerable cement; no apparent aperture”. Our specimens do not have a bordering flange, only a rim of smaller grains on the attachment surface. Aperture not visible. Its dimensions vary from 0.1 mm to 1.0 mm. It is the largest and most abundant attached foraminifer occurring in our samples. For the purpose of this study, we place “*Psammosphaera*-like” forms that contain a single larger grain in the genus *Hemisphaerammina*, although we note that intermediate forms between these two morphogenera do exist.

The Arctic specimens are most similar to *Hemisphaerammina bradyi* Loeblich and Tappan, described from the modern ocean (Loeblich & Tappan, 1957). It is a cosmopolitan form, distributed widely in cold regions (Culver & Buzas, 1980), that has a wide ecological tolerance (Schafer, Cole & Carter, 1981; Murray, 2006; Rodrigues et al., 2010; Rodrigues, Eichler & Eichler, 2013; Rodrigues, De Santis Braga & Eichler, 2015). It is found in shallow seas (Murray, 2006; Rodrigues et al., 2010) and from the deep ocean (Scott & Vilks, 1991; Scott et al., 2008). It is most common in regions with cold bottom water (Murray, 2006), and coarse sediment, high suspended matter, low pH, and low salinity (Rodrigues et al., 2010).

**Figure 5:**
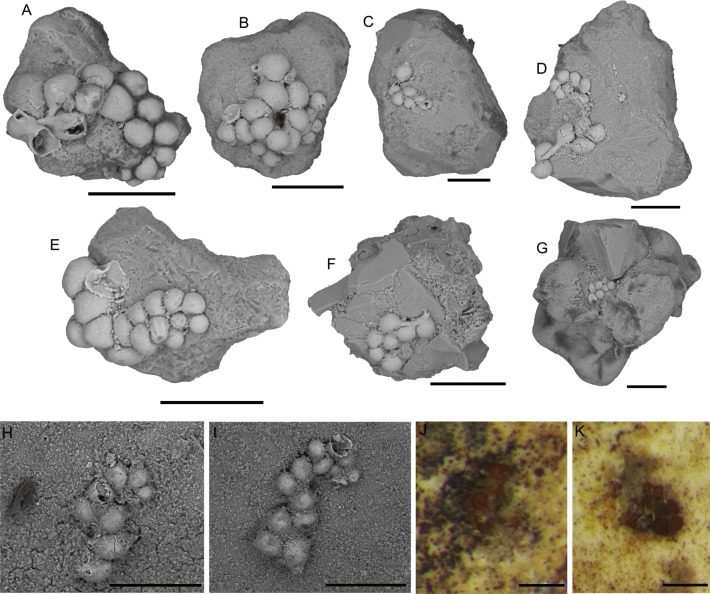
*Placopsilinella auriantiaca* Earland. (A–E) attached to clastic quartz, (F–G) attached to an agglutinated grain, (H–K) attached to the specimen of *C. striolatum*. Scale bar = 100 µm.

*6. Placopsilinella auriantiaca* Earland, 1934: Test attached, multi-chambered, forming a chain. The chamber arrangement is initially uniserial, may be coiled in a planispire, and later becoming biserial (loosely biserial, lax uniserial, or meandering) (Fig. 5). The latter part of the chain may again become uniserial or irregularly meandering. Specimens have up to 22 chambers with a diameter of 100–200 µm. Connections between chambers may be in the form of elongated stolons, and are areal in position. Chambers increase in size with ontogeny and become more pyriform, with longer stolons. Individual chambers are hemispherical in cross-section, and in top view may be pyriform in outline, with flat walls where they are in contact with adjacent chambers. The test wall was described as “proteinaceous with some ferruginous cement” by Loeblich & Tappan (1987), thin, reddish brown in colour, without agglutinated grains (Figs. 5J and 5K).

Our specimens display a more irregular chamber arrangement than the type specimens. Earland (1934) described forms consisting of up to 50 chambers from the Antarctic sector of the Southern Ocean.

*Placopsilinella auriantiaca* is widely distributed in the Boreal seas, and has been reported from the Arctic Ocean (Scott & Vilks, 1991; Wollenburg, 1992; Wollenburg & Mackensen, 1998; Husum et al., 2015), from the Labrador Sea (Schroeder-Adams & Van Rooyen, 2011); and the western North Atlantic (Kuhnt, Collins & Scott, 2000), and abyssal NE Atlantic (Stefanoudis, Bett & Gooday, 2016). Wollenburg described mass occurrences of *P. auriantiaca* in the Nansen Basin. It is also reported from the cool regions of the South Atlantic (Schumacher, 2001). Resig (1981) associated this species with increased primary production in the Peru Trench.

**Figure 6:**
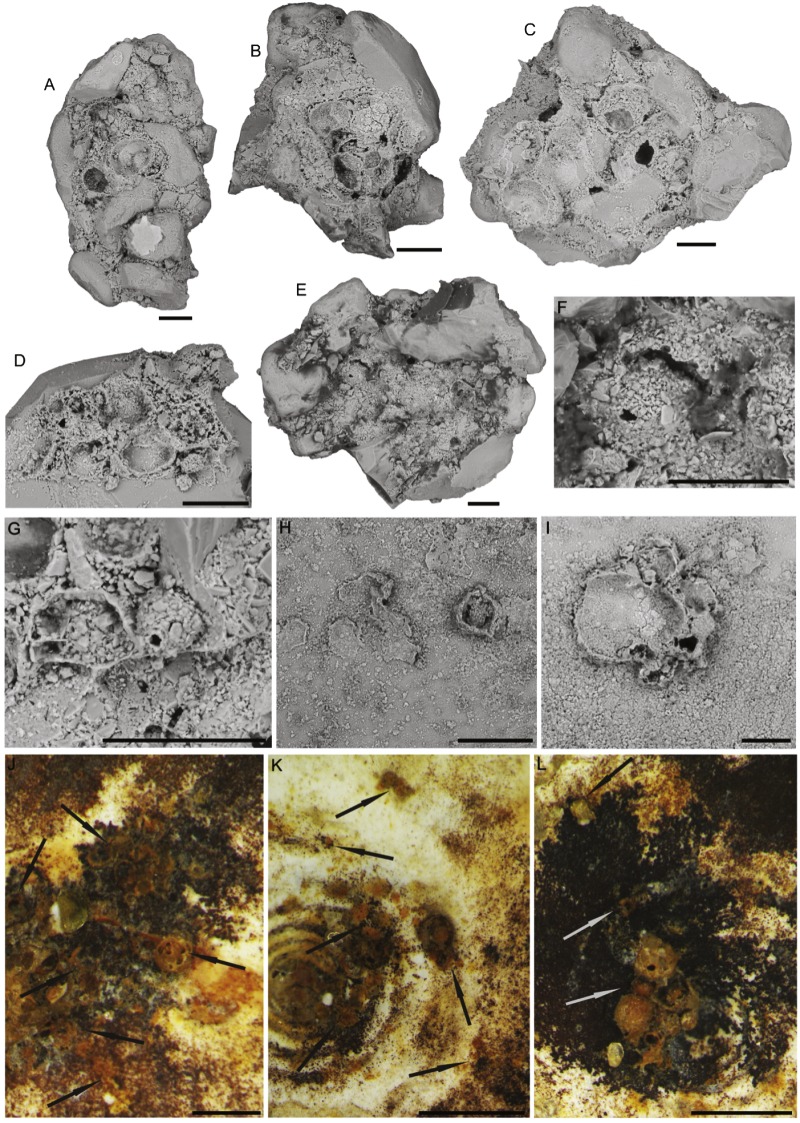
*Ammopemphix hemisphaericus* Waśkowska and Kaminski. (A–C), (E–F) Attached to an agglutinated grain, (F is after Waśkowska & Kaminski, 2018), (D) attached to clastic quartz, G–K attached to the specimen of *C. striolatum*. Scale bar: A–E, G–H = 100 µm, F = 200 µm, I–K = 500 µm.

*7. Ammopemphix hemisphaericus* Waśkowska & Kaminski, 2018: Clusters of small, attached chambers, often inhabiting the interiors of other attached foraminifera, but sometimes growing attached to mineral grains (Fig. 6). The chambers are hemispherical in shape, rounded externally, flat on the attachment surface. Their diameter varies from 20 to 150 µm. There is no connection between chambers. Mature specimens possess a single aperture, which is round and situated on a short collar. In small specimens the aperture is not visible. The test is yellow in colour and built of fine mineral grains and has a smooth surface. The test is delicate and is easily damaged. As a result, partial specimens are often found. Well preserved specimens are more likely to be preserved in depressions or cavities on the substrate grains. *Ammopemphix hemisphaericus* is one of the most common attached forms in the Arctic Pleistocene (Waśkowska & Kaminski, 2018).

The genus *Ammopemphix* is commonly observed in the polar regions (e.g., Wiesner, 1931; Cushman, 1948; Loeblich & Tappan, 1953; Majewski, 2005; Majewski, 2010). The Lomonosov Ridge is the type locality of the species *Ammopemphix hemisphaericus.*

**Figure 7:**
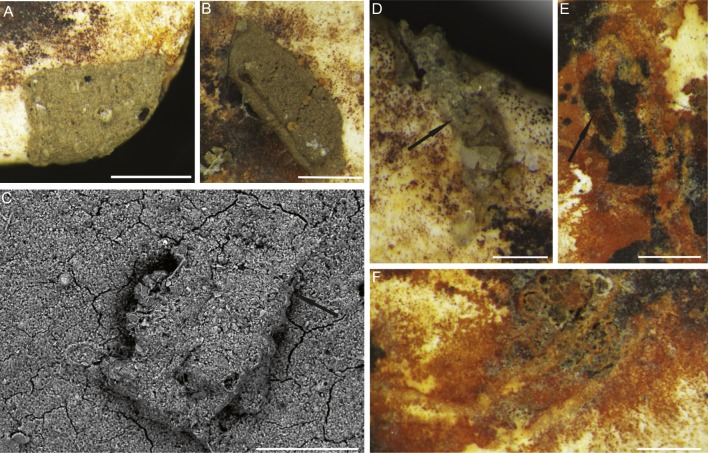
*Rhizammina-* like forms. (A–B), E—sp. 1, C–D, F—sp. 2, all attached to the specimen of *C. striolatum*. Remnants of *Ammopemphix hemisphericus* are seen in the upper part of F. Scale bar = 100 µm.

*8. Rhizammina*-like tubes: Two types of tubular forms are observed:

The first type (*Rhizammina* sp. 1) is a rather large form, with a tubular test that is 0.7 mm wide. This is a broad, thin, tubular species (Figs. 7A–7C) with a finely-agglutinated tubular wall comprised mainly of pelitic material with occasional larger grains. The test is collapsed. Only short fragments have been found, reaching a length of around 1 mm.

These tubular fragments resemble species of *Rhizammina* Brady, 1879 or *Septuma* Tendal and Hessler, 1977 that are known from the western North Atlantic (e.g., Kaminski, 1983, pl. 4, fig. 4). Modern tubular species sometimes attach themselves to a larger grain that is used as a holdfast. It is possible that the fragments observed in this study simply represent the attached part of the test.

The second type (*Rhizammina* sp. 2) is a tubular form that is sinuous, ca. 0.3 mm in diameter (Figs. 7C and 7F). The test is made of medium-size grains with occasional larger grains, and is round in cross section. The tests are only partially preserved, but the attachment surfaces are visible and extend for a length of about 1 cm. The tube does not appear to branch.

Murray & Renard (1891) mentioned “Rhizopod tubes” growing on metallic nodules, and Mullineaux (1987) noted *Rhizammina* aff. *algaeformis* Brady, 1879 on manganese nodules in the deep waters of the North Pacific. Forms similar to our *Rhizammina* sp. 2 were illustrated from the deep western North Atlantic by Kuhnt, Collins & Scott (2000, pl. 12, figs. 2, 3). Gooday, Goineau & Voltski (2015) illustrated meandering agglutinated tubes on manganese nodules from the Clarion-Clipperton Fracture Zone in the eastern equatorial Pacific, but these forms branch and form a mesh-like structure.

**Figure 8:**
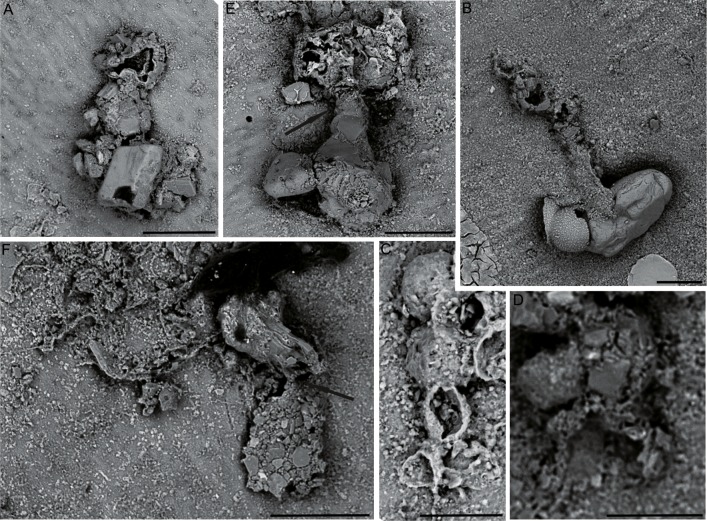
Uniserial forms. (A, C) *Subreophax*-like form, arrows indicate the necks between chambers. (B, D) *Hormosinelloides*-like form, (E–F) *Placopsilina*, all attached to the specimen of *C. striolatum*. Scale bar: A–D = 100 µm, E = 30 µm, F = 50 µm.

*9. Subreophax*-like forms: Test agglutinated, attached, consisting of a meandering series of hemispherical chambers (Figs. 8A and 8C). In our material, complete specimens were not observed, but the longest specimen is comprised of seven chambers, and the length of the chain reaches 0.5 mm. Chambers do not overlap, and should be best referred to as “pseudochambers”. The first three chambers initially increase in size rapidly, and subsequent chambers increase in size more slowly or not at all. Their diameter is in the range of 50–80 µm. A round aperture is situated on a short neck. Wall thin, coarsely or medium-agglutinated, a single grain thick. The wall is predominantly medium-grained, built of equidimensional grains, with the addition of some larger angular quartz particles. The initial chambers are built of smaller grains than the later chambers. Differs from the genus *Subreophax* Saidova, 1975 in its attached habitat.

The genus *Subreophax* is by definition free-living, however attached specimens are known to occur (Kuhnt, Collins & Scott, 2000). Mullineaux (1987) reported nodule-attached forms as *Hormosina* and *Reophax* from a site in the equatorial North Pacific. Kuhnt, Collins & Scott (2000) illustrated an attached form of *Subreophax aduncus* (Brady, 1882) and another species reported as *Subreophax* sp. from the deep waters of the western North Atlantic. Gooday, Goineau & Voltski (2015) described some attached forms as *Hormosina* sp. from the Clarion-Clipperton Fracture Zone in the eastern equatorial Pacific.

*10. Placopsilina* sp: Test agglutinated, attached, forming a meandering series of chambers (Figs. 8C and 8D). Chambers are small (10–50 µm in diameter) round or oval, and form chains. Initially the chamber arrangement is uniserial, and later the test branches to form additional chains that grow in parallel or appear to be braided. The chambers increase in size very slowly with ontogeny. The test wall is built of very fine quartz particles and has a smooth surface. An aperture is present at the end of the last chamber. Because our specimens are fragmentary, we are unable to verify the chamber arrangement in the early part of the test. According to the description of the genus by Cushman (1920), the initial portion should be close-coiled. Loeblich & Tappan (1987) stated that the early part is planispiral.

Our specimens resemble the species *Placopsilina confusa* Cushman, 1920, a well-known species in the modern ocean that is mostly reported from shallow waters (Hofker, 1983; Szarek, 2001; Försterra et al., 2005; Schönfeld et al., 2011), but is also known from deep waters (Cushman, 1920; Mullineaux, 1987; Schönfeld, 2002). A more common species is *Placopsilina bradyi* Cushman & McCulloch, 1939, reported by Loeblich & Tappan (1994) from the Timor Sea, by Szarek (2001) from the Sunda Shelf, and by Resig & Glen (1997) from the deep Pacific.

*11. Hormosinelloides*-like form: Test attached, consisting of chain of a pyriform compressed chambers (Figs. 8B and 8D). Wall coarsely and medium-agglutinated, of angular grains of varying dimensions, with the flat surfaces of the grains oriented toward the exterior, forming a smooth “pavement”. The species possesses a distinct elongated neck, but differ in the shape of the chambers. Our specimens are fragmentary, consisting of two chambers, but the attachment surface shows signs of another three chambers that have differing outlines than the two preserved chambers. One specimen likely represents a proloculus with the partial remains of a second chamber. In its initial part the test wall contains occasional larger grains. Their morphology the specimens most closely conform to the genus *Hormosinelloides* Zheng, 2001, in the fact that chambers appear to overlap, but it differs in its attached habitat.

It is possible that the genus has a partially attached mode of life. Scott & Vilks (1991) illustrated a specimen of *Hormosinelloides guttifer* (Brady, 1884) from the Arctic that is attached to a planktonic foraminifer. Even the type specimens preserved in the Brady Collection at the Natural History Museum pick up some larger agglutinated grains. Gooday, Goineau & Voltski (2015) list the genus *Hormosina* among forms that live attached to manganese nodules.

The genus *Placopsilina*, as well as the *Hormosinelloides*-like, and *Subreophax*-like forms were found attached to the specimen of *C. striolatum*.

**Figure 9:**
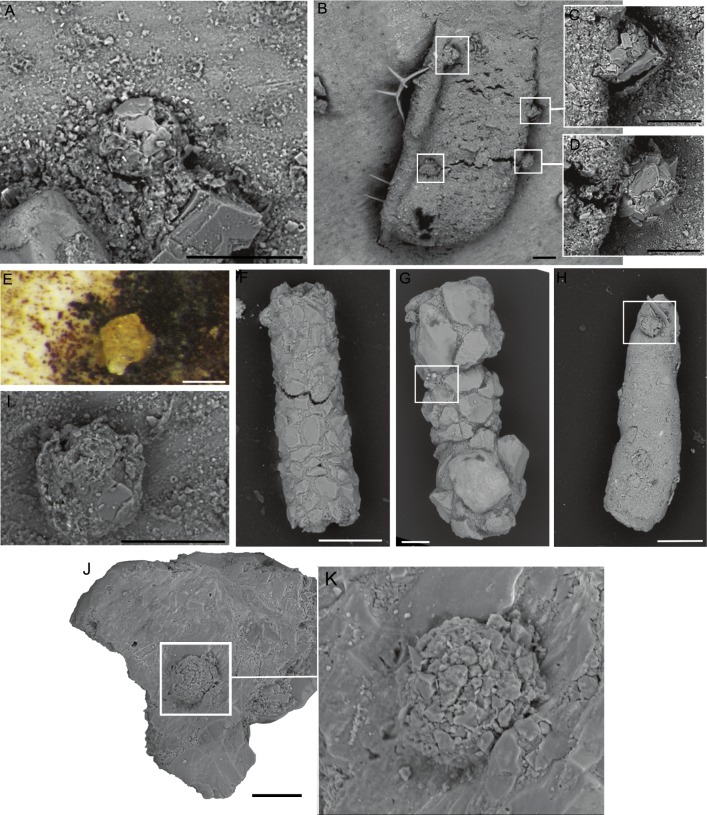
*Tholosina*-like forms. (A, E, I) Attached to the specimen of *C. striolatum*, (B–D), (F–H) attached to a tubular form, (J–K) attached to a mineral grain. Scale bar: A–B, D–H = 100 µm, C–D, E, I = 50 µm.

*12. Tholosina*-like forms: Singular small globular unilocular specimens with a circular to subcircular or oval outline (Fig. 9). Wall is medium agglutinated, comprised of quartz grains. In some specimens the grains appear to be arranged in a spiral manner around the uppermost central grain. Sometimes there is a single larger agglutinated grain. The diameter of the test varies from 30–60 µm. Aperture is usually not visible, but on one specimen a single areal opening is observed.

Our specimens are found attached to the specimen of *C. striolatum*, but most were observed attached to other agglutinated foraminifera (Fig. 9), such as *Rhizammina.* We also observed *Tholosina*-like forms attached to *Diffusilina*-like specimens and on mineral grains. They are relatively common in the Arctic Pleistocene.

Our specimens are morphologically similar to the species *Tholosina bulla* (Brady, 1881). *Tholosina* seems to be most commonly reported from the Polar seas. The modern species *Tholosina bulla* has been reported from the Arctic Ocean (Wollenburg, 1992), offshore Alaska (Loeblich & Tappan, 1953), Spitsbergen (Nagy, 1965; Majewski, Pawłowski & Zajączkowski, 2005; Majewski & Zajączkowski, 2007) and the Laptev Sea (Lukina, 2001), and from the western North Atlantic (Kuhnt, Collins & Scott, 2000). In the Southern Ocean it was reported from the Discovery stations (Earland, 1934; Earland, 1936) and from the Antarctic (Schröder-Adams, 1990). In the Peruvian upwelling zone it is commonly associated with phosphorite encrustations (Resig & Glen, 1997). Majewski, Pawłowski & Zajączkowski (2005) listed it as common at deep water stations in Spitsbergen fjords, and more seldom in shallow waters.

### Colonised substrates

In soft-sediment substrates the preferred substrate for attached agglutinated foraminifera are the mineral or lithic grains (ice-rafted detritus) that are significantly larger than the enclosing sediment. Their dimensions vary, but on the Lomonosov Ridge the average size is ca. 0.2 mm. In terms of composition, the substrate may be mineral or biogenic grains, and in the latter case these are often found encrusted with a dark metallic coating that we assume to be manganese oxide.

The abiotic (mineral or lithic) grains are in the medium to coarse sand range, and occasionally in the range of very coarse sand (on the Wentworth Scale). The quartz grains are usually rounded, though sharp sand grains with conchoidal fracture surfaces are also found (Figs. 4A–4D, 4F–4I, 5A–5G, 9A). The larger attached agglutinated foraminifera prefer smooth quartz grains, while the smaller forms apparently seek out crevices and depressions on the grains. The most common mineral colonised by attached agglutinated foraminifera is quartz, as this is the dominant mineral in the sand fraction of our samples. The species *Placopsilinella aurantiaca* and *Hemisphaerammina* (Figs. 4A–4D, 4F–4I, 5A–5E), are most often found on quartz grains. If the grain contains a cavern or depression, this is the preferred habitat for the pseudocolonial species *Ammopemphix hemisphaericus* (Figs. 4A and 6D).

The second category of substrate for attached agglutinated foraminifera are bioclasts. In the studied material, the most common substrate is the whole or broken tests of other species of agglutinated foraminifera (Figs. 2A, 9B–9D, 9F–9H, 5F–5G, 6A–6C, 6E–6G). Attached agglutinated foraminifera may be found within the interior of the chambers or nestled within cavities and crevices between agglutinated grains. The most common substrates are species of *Psammosphaera*, *Saccammina*, and *Reophax*. Less commonly the more finely agglutinated *Rhizammina*-like species serve as substrates. The most common species that grows attached to other agglutinated foraminifer is *Ammopemphix hemisphaericus.* The *Tholosina*-like forms are less common, and *Placopsilinella aurantiaca* is rarely observed attached to other foraminifera. Wollenburg (1992) estimated that about 10% of *Placopsilinella* specimens are attached to other foraminifera. Calcareous benthic foraminifera rarely serve as a substrate—in all our studied samples only one specimen of *Placopsilinella aurantiaca* was observed attached to a specimen of *Cassidulina laevigata* d’Orbigny, 1826. A few specimens of *Ammopemphix hemisphaericus* were observed attached to specimens of *Neogloboquadrina pachyderma* (Ehrenberg, 1861).

**Figure 10:**
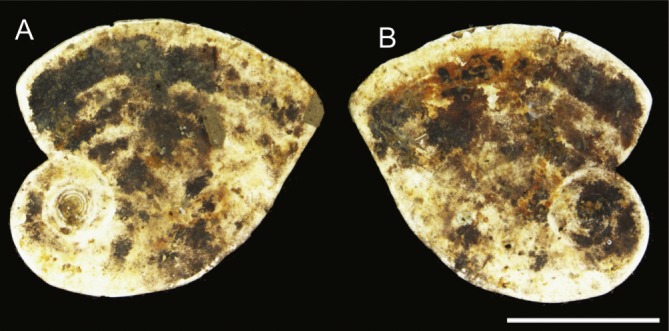
Specimen of *Cornuspirinoidesstriolatum* (Brady) with attached foraminifera. Scale bar = 1 cm.

Larger bioclasts are rare in the studied material, and may have been ice-rafted. In Sample PS87/100-1, CC, we found a relatively large (>10 mm) miliolid foraminifer *C. striolatum* (Fig. 10), which is partially encrusted with manganese or polymetalic oxide. This specimen was colonised by a whole range of attached agglutinated foraminifera. The colonisation took place on both sides of the specimen. All the various types of attached agglutinated foraminifera were observed. The original diversity of attached agglutinated foraminifera on this specimen could have been even higher, as some of the attached forms are only partially preserved. A large proportion of the smaller attached agglutinated foraminifera colonised depressions on the specimen of *C. striolatum* or are found within its depressed spiral suture. Larger species usually cement themselves to a flat surface.

## Discussion

Attached foraminifera are commonly reported elements of the modern foraminiferal assemblages in polar regions (Cushman, 1948; Höglund, 1947; Loeblich & Tappan, 1953; Loeblich & Tappan, 1957; Scott & Vilks, 1991; Wollenburg, 1992; Wollenburg, 1995; Wollenburg & Mackensen, 1998; Cornelius & Gooday, 2004; Majewski, Pawłowski & Zajączkowski, 2005; Majewski, 2010; Schroeder-Adams & Van Rooyen, 2011; Husum et al., 2015; Waśkowska & Kaminski, 2018). We also observe a diverse assemblage of attached foraminifera in the Pleistocene of the Arctic at depths between 951 and 2,772 m on the Lomonosov Ridge. Specimens are attached to larger mineral or biogenic particles that occur sporadically in otherwise soft substrates.

### Common morphotypes

The great majority of attached forms are simple agglutinated forms, mostly monothalamids, whereas only a single species (*Placopsilinella auriantiaca*) was found to have an organic wall. No attached calcareous benthic forms were observed. This is likely because of their low preservation potential in the largely noncalcareous sedimentary record of the Arctic.

The identified attached foraminifera are predominantly single-chambered monothalamids or primitive pseudochambered and multichambered agglutinated forms with simple wall structures. They either form flat sheets on the attachment surface or hemispherical elevations above the attachment surface. Most take the form of mats, sheets, roof-like structures, ribbons, or chains. Others form isolated domes. We have not observed any of the more complex forms that are known from manganese nodules, such as komoki, branched, or net-like forms (e.g., Mullineaux, 1987; Gooday, Goineau & Voltski, 2015). Interestingly, the attached multichambered trochamminids and lituolids known from modern shelf faunas (e.g., Debenay, 2012; Förderer & Langer, 2018) and the polar seas (e.g., Earland, 1934; Earland, 1936) are totally absent in our samples. Apparently in the extremely oligotrophic ice-covered glacial environment of the Central Arctic only the primitive simple agglutinated forms are able to survive.

### Microhabitat preferences

The most commonly observed attached form is *Ammopemphix hemisphericus*, which may be present singularly or as pseudocolonies (Waśkowska & Kaminski, 2018). The preferred microhabitat for this species was crevices and depressions between the grains of larger coarsely-agglutinated foraminifera, often in the interiors of chambers. Over 90% of the observed specimens were found attached to other agglutinated foraminifera (Figs. 6A–6C, 6G–6H). Rare specimens were found attached to quartz grains or on the surface of the specimen of *C. striolatum*, nestled in crevices or within the coil suture. Rare specimens were found attached to specimens of *Neogloboquadrina pachyderma.*

*Placopsilinella auriantiaca* was mostly found attached to quartz grains in our material (Figs. 5A–5E). The species occurs rarely on biogenic particles, such as on the tests of agglutinated foraminiferal and on the specimen of *C. striolatum*. In previous studies, the species has been reported mostly on quartz grains (Wollenburg, 1992), and less commonly on the tests of calcareous or planktonic foraminifera (Radford, Walker & Bowser, 2014), or on inorganic clasts (Earland, 1934; Cole, 1981; Kuhnt, Collins & Scott, 2000).

*Hemisphaerammina* sp. is one of the larger agglutinated species in our material. It almost exclusively attaches itself to a single large sand grain (Figs. 4A–4D, 4F–4I). Fortunately these are found in abundance as ice-rafted material in the Pleistocene of the Central Arctic. A single occurrence was observed on the specimen of *C. striolatum* (Fig. 4J).

*Tholosina*-like forms live attached to both abiotic grains and biogenic particles. In our material we mostly find them on biogenic grains, mostly on other agglutinated foraminifera such as finely agglutinated *Rhizammina* and on coarsely agglutinated *Reophax* and *Saccammina* (Figs. 9B, 9D–9F). They are also found on the specimen of *C. striolatum* (Figs. 9E and 9I) and on quartz grains (Fig. 9H). Cushman (1910), Cushman (1918) and Loeblich & Tappan (1953) found them attached to tubular agglutinated foraminifera, whereas Cushman (1948), Cushman & McCulloch (1939) and Majewski, Pawłowski & Zajączkowski (2005) reported them on *Hyperammina*, and Schröder-Adams (1990) illustrated specimens on quartz grains. Koho (2008) illustrated a specimen attached to a pteropod shell fragment.

The remaining types of attached agglutinated foraminifera, e.g., *Rhizammina*-like forms, *Diffusilina*–like, *Iridia*-like, ribbon-like, *Hormosinelloides*-like, *Subreophax*-like, and mat-like forms were only observed on the specimen of *C. striolatum.* The *Diffusilina*-like forms take advantage of cavities and depressions. Its roof-like cover is flush with the normal surface of the substrate (Fig. 2). The other forms attach to the flat surface and are elevated with respect to the substrate (Fig. 10).

**Figure 11:**
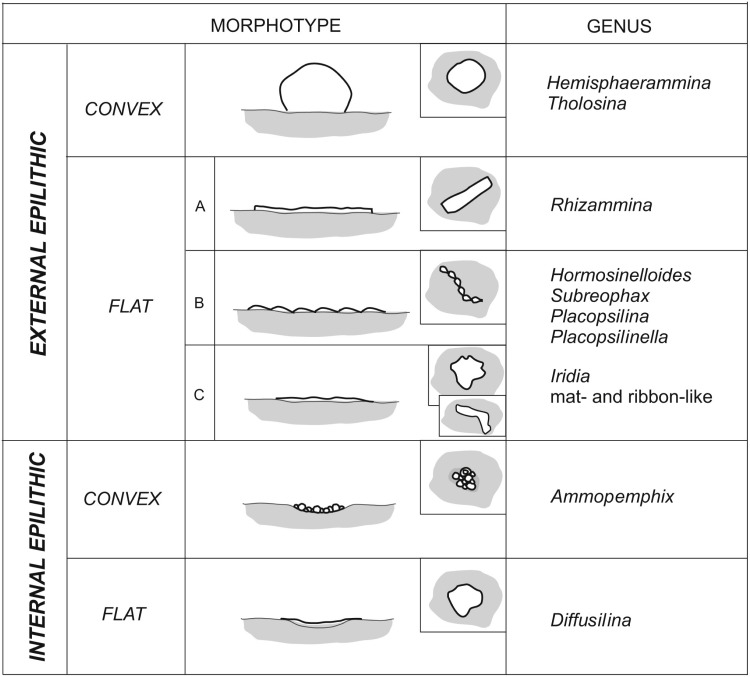
Morphotypes of Pleistocene attached foraminifera from the deep water Arctic environment.

The preferred microhabitats for the attached agglutinated foraminifera depend upon the dimensions of the attached species and the availability of appropriate pre-existing structures on the substrate. In the studied material we differentiate “internally epilithic” species from “externally eplithic” forms (Fig. 11). The majority of large hemispherical forms are externally epilithic—they sit on the exposed surface of the substrate and their tests form elevations on that surface. We distinguish forms that are high hemispheres, with globular or hemispherical shapes that are in contact with the substrate at the periphery of their tests. *Hemisphaerammina* sp. and *Tholosina bulla* belong to this group. A second group of externally epilithic forms are the flattened forms that only form low elevations on the substrate. In our material these comprise the meandering tubes and uniserial to multiserial pseudomultichambered or multichambered forms (Fig. 11, type A, B), and the elongated forms that form an irregular mat, e.g., *Iridia* (Fig. 11, type C). Flattened meandering forms are represented by the ribbon-like tubular forms such as *Rhizammina*-like forms, while the serial forms include *Placopsilina*, *Placopsilinella*, as well as the *Hormosinelloides*-like and *Subreophax*-like forms.

The internally epilithic forms settle into naturally-occurring dimples, depressions, and caverns on the surface of the substrate. These are small forms that prefer caverns or depressions, but may also outgrow their chosen space and exit their little cave. These include the forms that build a flat roof over their cavern, e.g., *Diffusilina*-type forms, as well as the pseudocolonies of *Ammopemphix hemispherica* (Fig. 11). In our material we did not observe any forms that use a substrate grain as a holdfast, i.e., attached dendritic forms that grow erect on the sea floor that are cemented at their base. Such forms are widely known from deep-sea environments in the modern ocean (e.g., Höglund, 1947; Tendal, 1972; Mullineaux, 1987; Gooday, Goineau & Voltski, 2015). Their absence in the Pleistocene may be due to their low preservation potential.

In our studied material, the majority of attached agglutinated foraminiferal would be placed in the epfaunal morphogroup based on their test morphology. These include the genera *Hemisphaerammina*, *Tholosina, Placopsilinella, Palcopsilina, Iridia, Diffusilina, Ammopemphix* and *Rhizammina.* However, the uniserial forms such as *Subreophax* and *Hormosinelloides* are usually placed in the infaunal category (Kaminski & Gradstein, 2005). The attached uniserial forms growing on the specimen of *C. striolatum* likely represent an exception, as they are associated with forms that undoubtedly epifaunal. Scott & Vilks (1991) illustrated a specimen of *Hormosinelloides* growing attached to a planktonic foraminifera, which suggests the specimen was growing in an epifaunal position. The change of habitat from infaunal to epifaunal would likely be caused by the extremely oligotrophic conditions of the glacial Arctic Ocean. Seasonal differences in the life position of deep sea benthic foraminifera have been observed in the modern ocean, for example at a 3,000 m deep station on the New Jersey margin, forms that were living in a shallow infaunal habitat in summer migrate up to the sediment surface during winter (Kaminski, Al Hassawi & Kuhnt, 1997), likely in search of food.

### Attached foraminiferal ecology

The most important environmental factor that decides the species composition of Arctic benthic foraminiferal assemblages appears to be the supply of organic matter to the sea floor (Altenbach et al., 1999; Wollenburg & Kuhnt, 2000). The ice-covered environment of the Central Arctic Ocean is extremely oligotrophic, especially during glacial periods (Wollenburg, 1995; Wollenburg, Kuhnt & Mackensen, 2001). The relative position of a suspension-feeding organism with respect to the substrate plays an important role in deciding whether that organism has access to organic particles carried in suspension (Altenbach, Unsöld & Walger, 1988; Schönfeld, 2002). Attached dendritic forms can access food particles carried in the benthic boundary layer, and other forms that have an elevated microhabitat likely have an advantage over those forms that grow flush with the substrate surface (Lutze & Thiel, 1989). A specimen of *Tholosina* growing on a tubular agglutinated foraminifera is likely taking advantage of the erect life position of its substrate host. As a consequence of their access to a greater food source, the externally epilithic forms such as *Hemisphaerammina* and *Placopsilinella* are larger and constitute the greatest numbers of specimens observed among the attached forms. The cavern-dwelling internally epilithic forms such as *Ammopemphix* may be exploiting a different trophic resource. These forms may be taking advantage of the bacterial flora that breaks down residual organic material present within the dead tests of foraminifera (Waśkowska & Kaminski, 2018), or bacteria associated with metalic oxide coatings on the substrate grains. A typical characteristic of the Arctic paleoenvironment is the presence of micronodules and polymetallic oxide coatings (mainly manganese oxide) on sediment grains (Stein, 2015). The colour of the coated grains varies from yellow to orange to dark brown and finally black. Such is the case with the specimen of *C. striolatum*. Dark coatings are commonly found covering the tests of Pleistocene agglutinated foraminifera—an example is the species *Haplophragmoides arcticus* (Kaminski, Waśkowska & Chan, 2016), which often displays dark coatings along its external sutures (Kaminski, Waśkowska & Chan, 2016, pl. 1, figs. 8, 9). Manganese nodules are known to be substrates for a large variety of attached agglutinated forms (e.g., Mullineaux, 1987; Gooday, Goineau & Voltski, 2015). A similar phenomenon is observed among the Pleistocene forms—the portion of the specimen of *C. striolatum* that is coated with manganese oxide displays a greater number and diversity of attached agglutinated foraminifera than the uncoated (white) areas (Fig. 10). The presence of polymetallic coatings—in actual fact the bacteria that produce them—apparently plays a role in recruiting and sustaining the attached agglutinated foraminifera.

Investigations of attached deep-sea foraminifera in other oceans suggest that as a whole the group is both opportunistic and able to colonise harsh environments. An attached trochamminid was found to colonise slate panels placed near deep-sea hydrothermal vents (Brönnimann, Van Dover & Whittaker, 1989). They may be found in the oxygen minimum zone (Resig & Glen, 1997), and in areas affected by strong bottom currents (Schönfeld, 2002). On the other hand, the attached group normally comprises only a small percentage of the total fauna in more diversified assemblages found under normal marine conditions. In the Holocene and especially during glacial periods the Arctic Ocean was extremely oligotrophic (e.g., Wollenburg, 1995; Wollenburg, Kuhnt & Mackensen, 2001), and therefore the benthic foraminifera had to adopt various life strategies in order to survive. The presence of ice cover lasting most of the year during interglacial periods and permanent ice cover during glacials caused the deep Arctic ocean to be extremely food-limited. In this case the dominant ecological factor influencing the composition of the benthic assemblage is likely to be the extreme oligotrophy. In the glacial Pleistocene the benthic foraminiferal assemblage is wholly agglutinated, and mainly comprised of only three species - *Reticulophragmium pusillum* (Brady), accompanied by *Haplophragmoides arcticus* Kaminski, Waśkowska and Chan, and *Trochammina lomonosovensis* Evans and Kaminski (Kaminski & Waśkowska, 2017). Other associated species such as tubular agglutinated forms, *Glomospira*, *Psammosphaera*, and other trochamminids are very rare. The attached forms such as *Ammopemphix*, *Hemispherammina*, *Tholosina,* and *Placopsilinella* comprise a significant component of these glacial assemblages. These forms have wide ecological tolerances in terms of bathymetry, oxygenation and food availability, are found in a wide range of environments, and are regarded to be opportunistic.

### Colonised substrates

The presence of medium to coarse-grained ice rafted sand was a necessary requirement, as this was the substrate most often colonised by attached forms. The second substrate was the presumably dead tests of other coarse-grained agglutinated foraminifera such as broken specimens of *Psammosphaera* found on the substrate surface. Attached foraminifera often colonised the interiors of these specimens, nesting into nooks and crannies between the agglutinated grains. Because they have settled on the (presumed) dead tests of other foraminifera, these attached forms are in fact eplithic, and simply utilised an available hard substrate. These are termed episkeletobionts (episkeletozoans) in the classification of Taylor & Wilson (2002) and Taylor & Wilson (2003). The microhabitat of the living individuals must play a role, for example *Reticulophragmium* and *Alveolophragmium* are regarded to be infaunal, and if their tests remain within the sediment after death, they would not be available for colonisation by the episkeletobionts. Although numerous calcareous bioclasts such as tests of planktonic foraminiferal, ostracods, and other shell fragments are present in the interglacial layers, they are rarely utilised by the attached agglutinated foraminiferal. The only exception is a single specimen of *Placopsilinella* attached to a specimen of *Cassidulina*, and a few colonies of *Ammopemphix* attached to specimens of *Neogloboquadrina pachyderma*. In their study of foraminiferal assemblage from the area of Novaya Zemliya, Korsun & Hald (1998) noted a correlation between the occurrence of attached forms and the presence of suitable clastic substrate particles. These authors did not find any correlation between attached forms and bathymetry. This also appears to be the case in our material from the Pleistocene of the Lomonosov Ridge.

## Conclusions

During the Pleistocene, the Central Arctic Ocean was host to a wide variety of attached agglutinated foraminifera that were able to exploit the extremely oligotrophic ice-covered glacial conditions prevalent at the time. The attached agglutinated foraminifera are mainly primitive monothalamous forms or simple chain-forming multichambered or pseudomultichambered forms, including representatives of the genera *Rhizammina, Hemisphaerammina, Ammopemphix , Diffusilina, Subreophax, Placopsilina, Placopsilinella, Hormosinelloides* and *Tholosina,* accompanied by mat-like and ribbon-like forms of uncertain taxonomic affinity. We separate two morphological types of attached agglutinated foraminifera, the externally epilithic forms that form elevations on the substrate surface, and the internally epilithic forms that colonise cavities and caverns within the substrate.

The epilithic agglutinated foraminifera colonised larger detrital grains (ice-rafted detritus) that are found on the otherwise muddy sea floor. The grains are mostly in size range of medium to coarse sand and are predominantly comprised of quartz, but bioclasts are also observed. The attached agglutinated foraminifera may colonise the presumably dead tests of other agglutinated foraminifera, or form encrustations on calcareous bioclasts. The different genera of attached agglutinated foraminifera display microhabitat preferences in terms of elevated or depressed microhabitats, which is likely related to their respective feeding strategies, i.e., whether they are suspension-feeders or bacteriovores.

## Additional Information and Declarations

### Data Availability

The following information was supplied regarding data availability: Microscope slides are housed in the European Micropalaeontological Reference Center at Micropress Europe, Kraków, Poland, with accession numbers 4/19c, 1-7.
